# Caveolin-1 regulation of Sp1 controls production of the antifibrotic protein follistatin in kidney mesangial cells

**DOI:** 10.1186/s12964-019-0351-5

**Published:** 2019-04-17

**Authors:** Neel Mehta, Dan Zhang, Renzhong Li, Tony Wang, Agata Gava, Pavithra Parthasarathy, Bo Gao, Joan C. Krepinsky

**Affiliations:** 1 Division of Nephrology, Department of Medicine, McMaster University, Hamilton, Canada; 2 Physiological Sciences Graduate Program, Health Sciences Centre, Federal University of Espirito Santo, Vitoria, Brazil; 3 St. Joseph’s Hospital, 50 Charlton Ave East, Rm T3311, Hamilton, ON L8N 4A6 Canada

**Keywords:** Caveolin-1, Mesangial cells, Follistatin, Sp1, Renal fibrosis

## Abstract

**Background:**

We previously showed that caveolin-1 (cav-1), an integral membrane protein, is required for the synthesis of matrix proteins by glomerular mesangial cells (MC). In a previous study to understand how cav-1 is involved in regulating matrix production, we had identified significant upregulation of the antifibrotic protein follistatin in cav-1 knockout MC. Follistatin inhibits the profibrotic effects of several members of the transforming growth factor beta superfamily, in particular the activins. Here, we characterize the molecular mechanism through which cav-1 regulates the expression of follistatin.

**Methods:**

Kidneys from cav-1 wild type and knockout (KO) mice were analyzed and primary cultures of MC from cav-1 wild-type and KO mice were utilized. FST promoter deletion constructs were generated to determine the region of the promoter important for mediating FST upregulation in cav-1 KO MC. siRNA-mediated down-regulation and overexpression of Sp1 in conjunction with luciferase activity assays, immunoprecipitation, western blotting and ChiP was used to assess the role of Sp1 in transcriptionally regulating FST expression. Pharmacologic kinase inhibitors and specific siRNA were used to determine the post-translational mechanism through which cav-1 affects Sp1 activity.

**Results:**

Our results establish that follistatin upregulation occurs at the transcript level. We identified Sp1 as the critical transcription factor regulating activation of the FST promoter in cav-1 KO MC through binding to a region within 123 bp of the transcription start site. We further determined that the lack of cav-1 increases Sp1 nuclear levels and transcriptional activity. This occurred through increased phosphoinositide 3-kinase (PI3K) activity and downstream protein kinase C (PKC) zeta-mediated phosphorylation and activation of Sp1.

**Conclusions:**

These findings shed light on the transcriptional mechanism by which cav-1 represses the expression of a major antifibrotic protein, and can inform the development of novel antifibrotic treatment strategies.

**Electronic supplementary material:**

The online version of this article (10.1186/s12964-019-0351-5) contains supplementary material, which is available to authorized users.

## Background

Mesangial cells (MC) are specialized pericytes involved in the production and secretion of mesangial matrix within glomeruli of kidneys [1–3]. The mesangial matrix serves to anchor MC and provide structural support and integrity to the surrounding glomerular capillaries [1–3]. Under homeostatic conditions, MC secrete matrix that is composed of collagens, laminin and fibronectin [1, 4–6]. The activation and transition of MC to a more secretory myofibroblastic phenotype has been established to be an early fibrogenic response in kidney disease of varying etiology, including that due to diabetes and hypertension [1–7].

Caveolae are small (50–100 nm) glycosphingolipid- and cholesterol-enriched omega-shaped invaginations of the plasma membrane that are involved in mediating a wide array of signaling transduction events [8–10]. Through compartmentalization of signaling proteins, caveolae can either positively or negatively mediate signal transduction [8–10]. The caveolin (cav) gene family consists of three proteins, cav-1, cav-2 and cav- 3. Cav-1 and cav-2 are ubiquitously expressed, whereas cav-3 is limited to skeletal muscle, diaphragm, and heart [8–10]. Formation of caveolae requires cav-1, a 21–24 kDa integral membrane protein [8–10]. We have previously shown that the ability of MC to produce matrix proteins both basally and in response to profibrotic stimuli such as transforming growth factor beta beta 1 (TGFβ1), mechanical stress, and high glucose is dependent on cav-1 expression [11–13]. Importantly, diabetic mice lacking cav-1 are protected against mesangial matrix expansion and the development of glomerular sclerosis [11]. Strong upregulation of cav-1 has also been demonstrated in rodent models of chronic kidney disease and diabetic nephropathy [10, 14]. These studies support a profibrotic role for cav-1/caveolae in kidney fibrosis.

Clinically targeting cav-1 in vivo is challenging [10]. Thus, to better understand how cav-1 elimination reduces matrix production in MC, and more importantly, to identify potential novel therapeutically applicable targets that can be exploited to overcome the difficulties associated with directly targeting cav-1, our lab previously identified and measured the expression of potential anti-fibrotic candidates that are altered in cav-1 deficient MC. Of primary interest, we identified significant upregulation of follistatin (FST), an anti-fibrotic factor, in MC lacking cav-1.

FST is an ubiquitously expressed and secreted glycoprotein that binds to and neutralizes the profibrotic and proinflammatory actions of several TGFβ superfamily members, with greatest activity against activins [15, 16]. We and others have shown that FST acts as a strong antifibrotic agent in various organs, including the kidneys in models of obstructive kidney damage and diabetic nephropathy (Aoki et al., [17]; Patella et al.,[18]; Maeshima et al.,[19]; Zhang et al., Kidney Int, submitted).

The molecular mechanism through which FST is regulated by cav-1 in glomerular MC is as yet unknown. Here, we show that signaling through the phosphoinositide 3-kinase (PI3K), protein kinase C zeta (PKCζ) and Sp1 signaling pathway is augmented in cav-1 deficient MC to increase the transcriptional regulation of FST. These findings shed insight into the molecular mechanism through which cav-1 regulates the expression of FST and provide important knowledge that can inform the development of antifibrotic treatment strategies for chronic kidney disease.

## Methods

### Cell culture

Primary mouse MC were isolated from male cav-1 wild-type (WT) and cav-1 knockout (KO) B6129SF1/J mice (Jackson Laboratory) using Dynabeads (Invitrogen). Briefly, mice were perfused with magnetic Dynabeads, kidneys were harvested and digested by collagenase and glomeruli were collected using a magnet. Isolated glomeruli were washed with HBSS, resuspended in Dulbecco’s modified Eagle’s medium supplemented with 20% fetal bovine serum (Invitrogen), penicillin (100 μg/ml) and streptomycin (100 μg/ml) at 37 °C in 95% O_2_, 5% CO_2_. MC were grown out, with passages 7–14 used for experiments. MC were serum deprived in 0.5% FBS 24 h prior to all treatments unless otherwise stated. Drugs/reagents used in the study are provided in Additional file 1: Table S1.

### Transfection

MC at 60–70% confluence were transfected (0.5 μg luciferase plasmid with 0.05 μg β-galactosidase or 1-2 μg protein expression plasmid) using Effectene (Qiagen) as per the manufacturer’s recommendation. siRNA-mediated knockdown was achieved using RNAiMAX (Thermo Fisher Scientific) as per the manufacturer’s recommendation. Plasmids and siRNA used in the study are provided in Additional file 1: Table S2.

### Luciferase assay

MC lysis was achieved using Reporter Lysis Buffer (Promega) as per the manufacturer’s recommendation. Luciferase activity was measured on clarified cell lysate using the Luciferase Assay System (Promega) with a luminometer (Junior LB 9509, Berthold). β-galactosidase activity, used to normalize for transfection efficiency, was measured in clarified cell lysates using the β-Galactosidase Enzyme Assay System (Promega) with a plate reader absorbance set at 420 nm (SpectraMax Plus 384 Microplate Reader, Molecular Devices).

### Protein extraction, immunoprecipitation and immunoblotting

MC cell lysis and protein extraction were carried out as described previously [20]. Briefly, cell lysates were centrifuged (15,000 rpm, 10 min, 4 °C), supernatant was collected and protein concentration quantified. For immunoprecipitation experiments, cells were lysed, clarified and equal amounts of lysate were immunoprecipitated using 1 μg primary antibody (18 h, 4 °C), followed by incubation with protein-G–agarose slurry (2 h, 4 °C). Cell protein lysates (10 μg–50 μg) and immunoprecipitated products (total yield) were separated on SDS-PAGE for subsequent immunoblotting. Antibodies used in the study are provided in Additional file 1: Table S3.

### Quantitative-real time PCR

RNA from MC was extracted using Ribozol RNA Extraction Reagent (Amresco) as per the manufacturer’s recommendation, with 1 μg of RNA reverse transcribed into cDNA using qScript cDNA SuperMix Reagent (Quanta Biosciences). Quantitative real-time PCR was carried out using the Power SYBR Green PCR Master Mix (Thermo Fisher Scientific) on the Applied Biosystems ViiA 7 Real-Time PCR System (Thermo Fisher Scientific). mRNA expression and fold changes were calculated using the ΔΔC_T_ method, where 18S was used as the endogenous control. Primer sequences used in the study are provided in Additional file 1: Table S4.

### ChIP

At endpoint, MC were cross-linked using formaldehyde (10 min, RT), neutralized using 1 M glycine (pH 2.2) (5 min, RT), resuspended in ice-cold PBS containing protease inhibitors and centrifuged (13,000 rpm, 5 min, 4 °C). The cell pellet was resuspended in nuclear extraction buffer (20 mM HEPES pH 7.9, 25% glycerol, 420 mM NaCl,1.5 mM MgCl_2_,0.2 mM EDTA, protease inhibitors), incubated on ice (20 min) and centrifuged (13,000 rpm, 10 min, 4 °C). The resulting nuclear pellet was resuspended in Breaking Buffer (50 mM Tris-HCl pH 8.0, 1 mM EDTA, 150 mM NaCl, 1% SDS, 2% Triton X-100, protease inhibitors), sonicated 6x3s, and Triton Buffer added (50 mM Tris-HCl pH 8.0, 1 mM EDTA, 150 mM NaCl, 0.1% Triton X-100). 10% of the original aliquot was collected (input) and the rest used for immunoprecipitation for Sp1 and mouse IgG (as described above). Immunoprecipitated samples were washed 3x in Triton Buffer. SDS Buffer was then added (62.5 mM Tris HCl pH 6.8, 200 mM NaCl, 2% SDS, 10 mM DTT, 2 μl of proteinase K (40 mg/ml)) and samples incubated (18 h, 65 °C) to reverse crosslinking. DNA was isolated using phenol/chloroform extraction and resuspended in dH_2_O. qRT-PCR was used, as described above, to amplify the purified DNA using primers specific to the Sp1 binding site located within -123 bp of the mouse FST (mFST) promoter. Ct values were evaluated across multiple replicate experiments using the %input method, where %input = 100*2^(Adjusted input - Ct (IP))^.

### Cloning

The full-length mouse FST luciferase (−luc) construct, mFST4-FL-luc, (obtained from Dr. Jeong Yoon) containing the mouse FST promoter with exon 1 and intron 1 was digested with KpnI and NheI. The resulting product was inserted into a linearized pGL3-luc vector in order to generate a construct which lacks intron 1 and most of exon 1 (+ 20 bp is included, where transcription start site = + 1). The resulting plasmid hereafter is referred to as mFST4-luc. mFST4-luc was used to generate mFST promoter deletion constructs using the primer sequences listed in Additional file 1: Table S5.

Two Sp1 binding sites (CCGCCC) localized within the mFST4–123 bp promoter were deleted in order to generate mFST4Δintron1-123ΔSp1-luc. Briefly, oligonucleotides coding the FST-123 promoter sequence lacking the two Sp1 binding sites along with the KpnI (5′) and NheI(3′) digestion sites were synthesized, annealed, and ligated into pGL3-Basic luc. All sequences synthesized for cloning are listed in Additional file 1: Table S5. All generated constructs were confirmed by sequencing (Mobix Lab, McMaster University).

### PI3K activity assay

A plasmid encoding a Venus-tagged pleckstrin homology (PH) domain of Akt (PH-Akt-Venus), a gift from Dr. Narasimhan Gautam (Addgene plasmid # 85223), was transfected into MC. Plasma membrane localization of the PH domain of Akt was used as a live phosphatidylinositol 3,4,5-trisphosphate (PIP3) sensor for assessing PI3K activity [21]. Briefly, 24 h following transfection, cav-1 WT and KO MC were incubated with wheat germ agglutinin (WGA) Alexa Fluor-594 Conjugate (Thermo Scientific) in HBSS (2 μg/ml, 10 min, 37 °C) to delineate the plasma membrane. After plasma membrane labeling, MC were washed and images were taken using a fluorescein (ex490nm/em525nm) and rhodamine (ex550nm/em620nm) filter sets (EVOS FL Cell Imaging System, Thermo Fisher Scientific). Image J, in conjunction with the co-localization finder plugin (https://imagej.nih.gov/ij/plugins/colocalization-finder.html), was used to create colocalization masks and quantify the percent localization of PH-Akt-Venus to the cell membrane.

### Immunohistochemistry/immunocytochemistry

Cav-1 WT and KO B6129SF1/J mice were sacrificed and perfused with cold PBS in accordance with principles of laboratory animal care and McMaster University and Canadian Council on Animal Care guidelines. For immunohistochemistry, 4 μm FFPE kidney sections were deparaffinized, endogenous peroxidase activity was blocked, and heat-induced epitope retrieval was carried out for immunohistological staining. Briefly, tissues were blocked with 5% horse serum and incubated in primary antibody overnight at 4 ͦ C. Tissues were then incubated with biotinylated secondary antibodies (Vector Labs) (30 min, room temperature) and then incubated with streptavidin/peroxidase (30 min, room temperature) (Vector Labs). Chromogenic color development was carried out using Nova Red (Vector Labs), followed by counterstaining using Gill’s hematoxylin (Sigma), and mounting in a xylene based mounting media (Permount; Thermo Scientific). All micrographs were captured at × 200 and × 400 magnification using the BX41 Olympus microscope. The total percentage of positive area (signal) within the kidneys was measured using ImageJ.

Serum deprived MC plated in an 8-well chamber slide were used for ICC. Cells were fixed in 4% paraformaldehyde, permeabilized in 0.2% Triton X-100, blocked in 1% BSA/3% donkey serum, and incubated with primary antibodies overnight at 4 ͦ C. Cells were then incubated with Alex-Fluor (Thermo Scientific) conjugated secondary antibodies (30 min, room temperature, dark), and mounted and counterstained using a DAPI-containing fluorescent mounting media (Vector Labs). All ICC micrographs were captured using the fluorescein (ex490nm/em525nm) and DAPI (ex350nm/em470nm) filter sets (EVOS FL Cell Imaging System, Thermo Fisher Scientific). Mean fluorescence intensity within the nucleus, delineated using DAPI and/or total cellular expression examined under the appropriate fluorescence filter sets was measured using ImageJ.

### Statistical analysis

Statistical analyses were performed using GraphPad Prism 6. A Student’s *t*-test or one-way ANOVA was used to determine statistical significance between two or more groups, respectively. Post hoc significance of pairwise comparisons was assessed using Tukey’s HSD. A *p*-value < 0.05 (two-tailed) was considered significant. Data are presented as mean ± SEM. The number of experimental repetitions (*n*) is indicated in the figure captions.

## Results

### Cav-1 regulation of FST occurs at the transcript level in MC

**Fig. 1:**
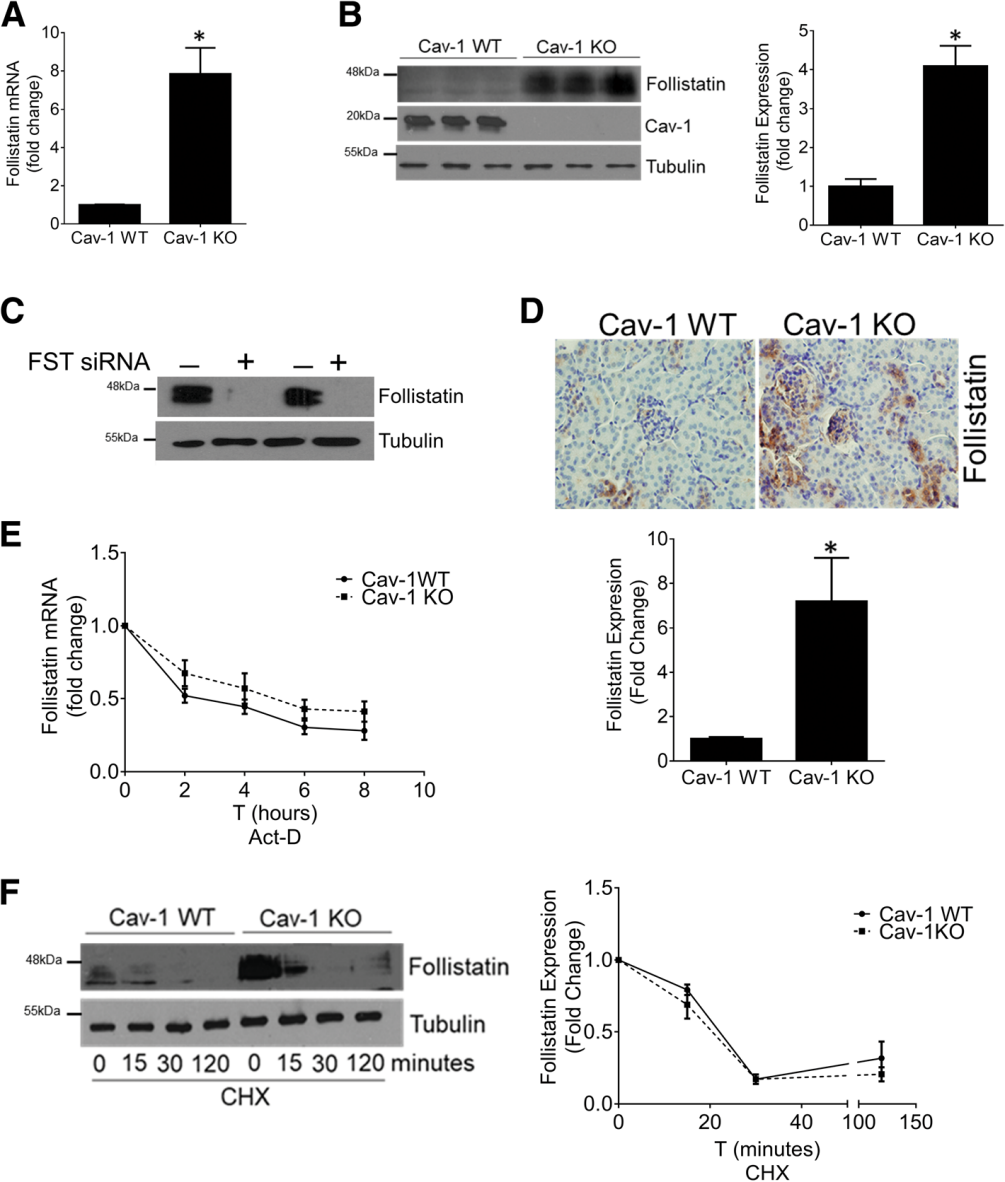
Cav-1 transcriptionally represses FST. In primary cav-1 KO MC, FST expression was increased at both the transcript level (*n* = 12, *p < 0.05) (**a**) and protein level (*n* = 9, *p < 0.05) (**b**). **c** Immunoblotting after FST downregulation with siRNA showed the specificity of the FST antibody in cav-1 KO MC (*n* = 2). **d** FST was elevated in the kidneys of cav-1 KO mice (*n* = 4 mice, *p < 0.05, representative micrographs shown). **e** MC were treated with the transcriptional inhibitor actinomycin D (1 μg/ml), and FST transcript assessed at the indicated times. FST mRNA stability did not differ between cav-1 WT and KO MC (*n* = 5). **f** Cav-1 WT and KO MC were treated with the translational inhibitor cycloheximide (10 μg/ml), and FST protein assessed at the indicated times. FST protein stability was not altered by cav-1 KO (*n* = 3)

We have identified FST as a significantly upregulated gene in cav-1 deficient MC compared to their wild-type (WT) counterparts. Figure 1 shows elevated FST expression at the mRNA (Fig. 1a) and protein level (Fig. 1b) in cultured primary cav-1 KO MC. It should be noted that the FST antibody detects two main bands in MC. Using FST siRNA, we confirm that these are both FST (Fig. 1c). This is likely due to the presence of different isoforms and/or differentially glycosylated forms of the protein [22–25]. Increased FST expression is also seen in the kidneys of mice lacking cav-1 compared to WT mice (Fig. 1d). Confirming the anti-fibrotic properties of FST in MC, TGF-β1-mediated extracellular matrix protein (ECM) production was blunted by the addition of recombinant FST in cav-1 WT MC (Additional file 2: Figure S1A). Conversely, FST downregulation using siRNA significantly augmented TGF-β1-medited ECM production in cav-1 KO MC (Additional file 2: Figure S1B). These results confirm that FST inhibits TGF-β-induced ECM production in MC.

To identify potential avenues by which cav-1 regulates FST expression, we began by examining stability of the FST transcript in cav-1 WT and KO MC. Using actinomycin D to stop de novo transcription, we observed that FST mRNA has a half-life of about 4 h which was unaffected by cav-1 expression (Fig. 1e). We next assessed whether FST protein stability was affected by cav-1. Using cycloheximide to prevent de novo protein synthesis, we determined that FST has a rapid turnover rate, which is also unaffected by cav-1 expression (Fig. 1f). The increased transcript and protein levels seen in cav-1 KO MC are thus not a result of increased post-transcriptional mRNA stability or post-translational protein stability.

**Fig. 2:**
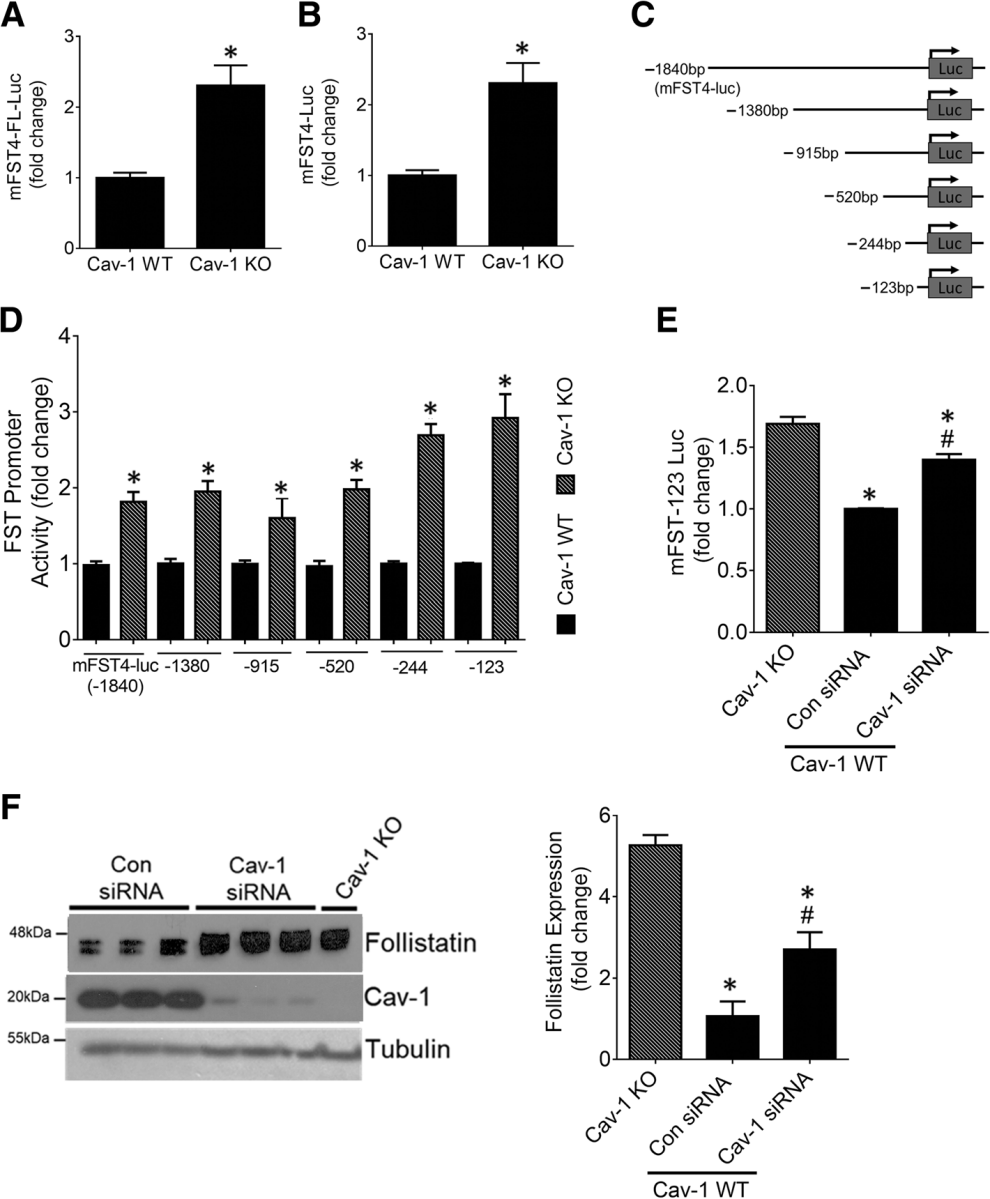
Cav-1 regulates activity of the FST promoter. Cav-1 WT and KO MC were transfected with (**a**) the full-length FST promoter luciferase construct or (**b**) the full-length FST promoter luciferase construct lacking intron 1 and exon 1. KO MC exhibited significantly elevated transcriptional activity of both constructs (*n* = 6, *p < 0.05). **c** Graphical representation of the synthesized FST promoter deletion constructs. **d** Cav-1 WT and KO MC were transfected with the FST promoter deletion constructs shown in (C). Transcriptional activation of all constructs was elevated in cav-1 KO MC (*n* = 6–20, *p < 0.05 vs WT for each construct). **e**, **f** Cav-1 knockdown in WT MC significantly increased the transcriptional activation of the -123 bp FST promoter (**e**) and FST protein expression (**f**) compared with control siRNA-transfected KO MC (for both, *n* = 6, *vs KO, #vs WT con siRNA, *p* < 0.05)

We next sought to determine whether cav-1 regulates FST expression at the transcriptional level. The mouse FST promoter has been previously characterized [26]. Using a mouse FST promoter luciferase reporter construct, we assessed whether FST promoter activity was differentially regulated in cav-1 WT and KO MC. Here, we found significantly elevated transcriptional activity in cav-1 KO MC of both mFST4-FL-luc, which also contains intron 1 and exon 1 of FST, and mFST4-luc in which these are removed (Fig. 2a and b). Since removal of intron 1 and exon 1 did not affect cav-1 KO upregulation of FST promoter activity, we used mFST4-luc as a template to generate promoter deletion constructs ranging from -1840 bp to -123 bp upstream of the transcription start site (TSS) (Fig. 2c). Here, we surprisingly found significantly elevated transcriptional activity of all promoter deletion constructs in cav-1 KO MC (Fig. 2d). These data illustrate that transcriptional regulatory elements residing between -123 bp and the TSS of the mouse FST promoter are critical for the upregulation of FST that is observed in cav-1 KO MC. Next, using siRNA-mediated cav-1 downregulation in cav-1 WT MC, we confirmed that cav-1 was directly involved in the regulation of FST promoter activity (Fig. 2e) and protein expression (Fig. 2f). However, the relative increase in FST transcription and expression upon cav-1 downregulation with siRNA was not as effective in comparison to the elevation seen in cav-1 KO MC. This is likely due to differences in the degree of cav-1 suppression between these two approaches.

### Cav-1 transcriptionally regulates FST through Sp1

**Fig. 3:**
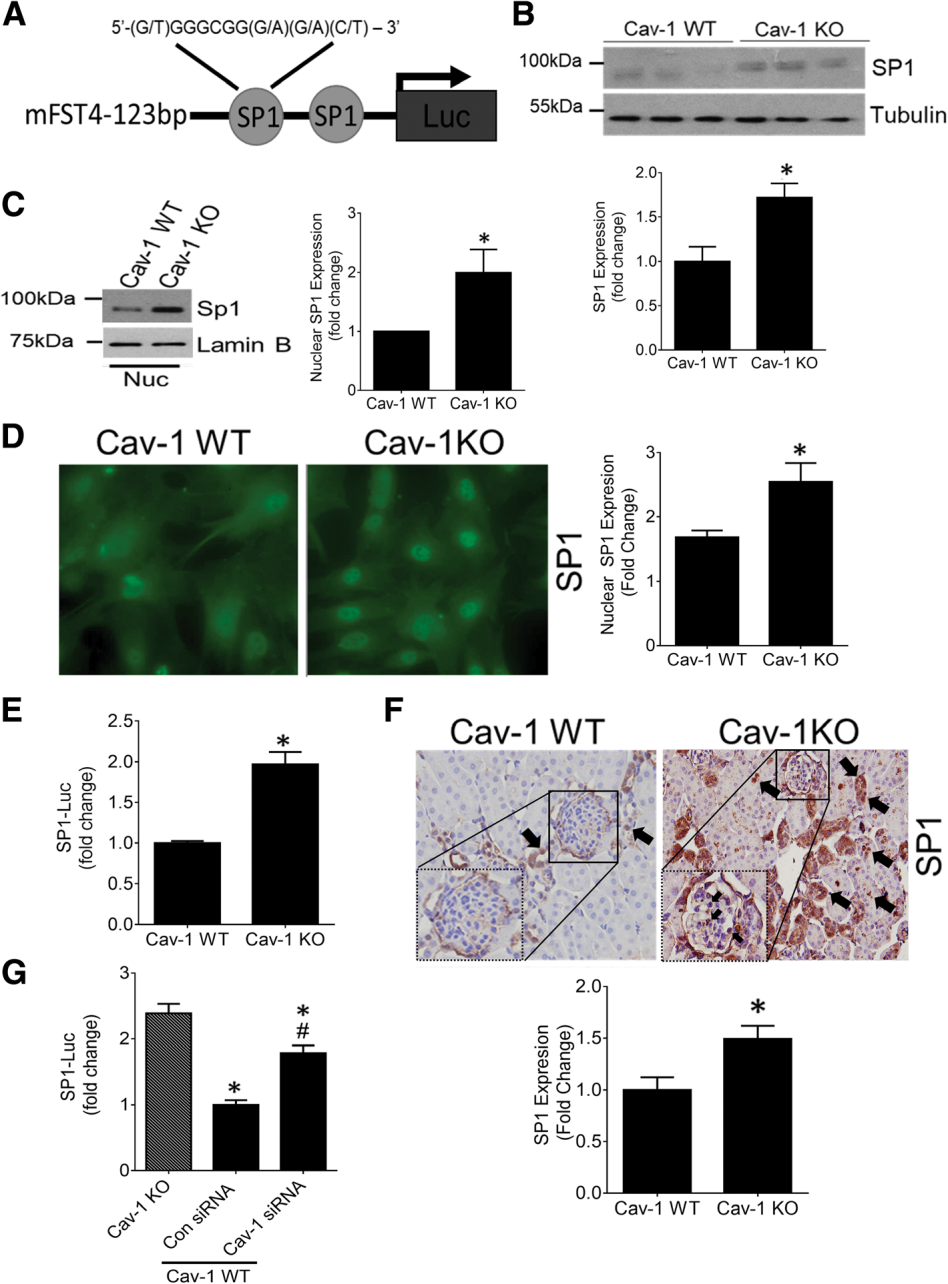
Sp1 expression and activity are elevated by cav-1 deletion. **a** Predicted Sp1 transcription factor binding sites within the -123 bp region of the FST promoter are shown. Sp1 protein expression was significantly increased in cav-1 KO compared to WT MC as assessed by western blotting of total cell lysate (*n* = 12, *p < 0.05) (**b**) and nucleus (*n* = 5, *p < 0.05) (**c**), as well as by immunofluorescence microscopy (*n* = 5, *p < 0.05, representative micrographs shown) (**d**)**. e** Sp1 activity, as assessed by the Sp1 reporter construct Sp1-luc, was elevated in cav-1 KO MC (*n* = 16, *p < 0.05). **f** Sp1 expression and nuclear localization (arrows) were elevated in the kidneys of cav-1 KO mice, seen in both glomeruli (magnified glomerular area shown within dotted box) and tubules (*n* = 3 mice, *p < 0.05, representative micrographs shown). **g** Cav-1 knockdown in WT MC significantly increased Sp1 activity compared with control siRNA-transfected KO MC (*n* = 6, *vs KO, #vs WT con siRNA, *p* < 0.05)

Having established a transcriptional effect of cav-1 on the -123 bp FST promoter, we next screened for transcription factor regulatory element(s) located within this region. Putative transcription factor binding sites within the mFST promoter were identified using MatInspector and PROMO [27]. Interestingly, we identified two Sp1 binding sites (CCGCCC) in this region (Fig. 3a), with Sp1 having been shown to regulate FST promoter activity in intestinal epithelial cells [28]. We thus assessed its role in mediating FST upregulation in cav-1 KO MC. We first determined whether Sp1 levels are altered in these cells. To this end, we found elevated Sp1 expression in cav-1 KO MC (Fig. 3b). This was associated with increased Sp1 nuclear presence, as assessed by immunoblotting (Fig. 3c) and immunofluorescence (Fig. 3d), as well as increased transcriptional activity as measured using a Sp1-target sequence binding luciferase (Fig. 3e). Similar increases of Sp1 expression and nuclear localization were seen in both glomeruli and tubules of cav-1 KO mice (Fig. 3f). Next, using siRNA-mediated cav-1 downregulation in WT MC, we confirmed the regulation of Sp1 activity by cav-1 (Fig. 3g).

**Fig. 4:**
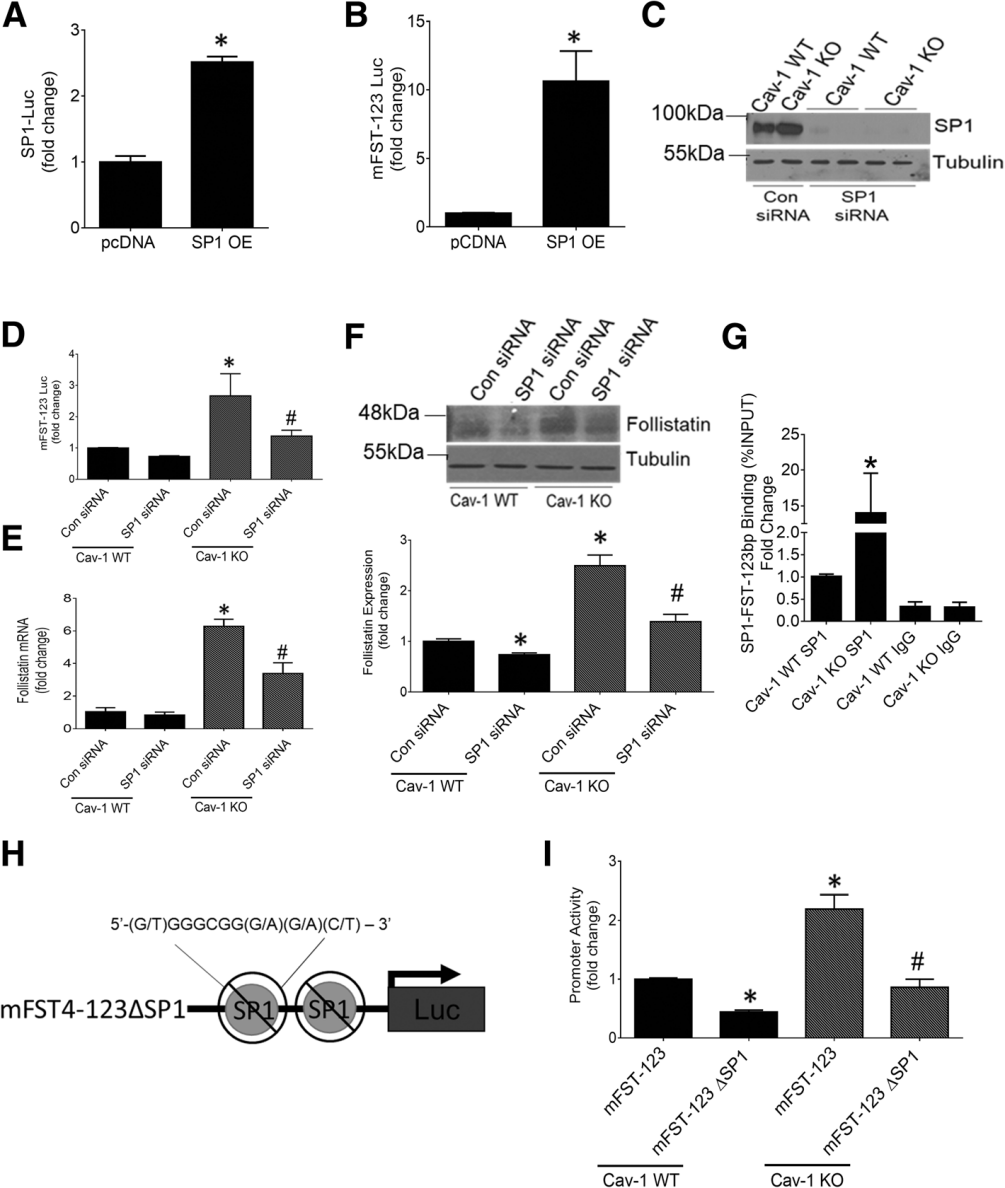
Sp1 binds the -123 bp region of the FST promoter to regulate its activity. Expression of constitutively active Sp1 in cav-1 WT MC increased activity of both the Sp1 reporter construct (**a**) (*n* = 3, *p < 0.05) and mFST-123-luc (**b**) (*n* = 6, *p < 0.05). **c** Effective siRNA-mediated Sp1 knockdown in cav-1 WT and KO MC was confirmed by immunoblotting. **d** Sp1 knockdown abolished the increased transcriptional activity of the -123 bp FST promoter in cav-1 KO MC, with little effect in WT cells (*n* = 6, *vs WT con siRNA, #vs KO con siRNA, *p* < 0.05). **e** Sp1 knockdown repressed the elevation in FST mRNA expression in cav-1 KO MC (*n* = 3, *vs WT con siRNA, #vs KO con siRNA, *p* < 0.05). **f** Similar effects of Sp1 knockdown on FST protein expression were seen (*n* = 4, *vs WT con siRNA, #vs KO con siRNA, *p* < 0.05). **g** Sp1 binding within the -123 bp region of the FST promoter at the predicted Sp1 binding sites was significantly elevated in cav-1 KO MC as assessed by ChIP (*n* = 14, *p* < 0.05). **h** Graphical representation of the deletion of the two predicted Sp1 binding sites within the -123 bp promoter region of FST (mFST4–123ΔSp1-luc). **i** Deletion of the two Sp1 binding sites attenuated transcriptional activity of mFST4–123-luc in cav-1 WT and normalized activity in cav-1 KO MC to levels seen in WT cells (*n* = 9, *vs WT mFST-123-luc, #vs KO mFST-123-luc, *p* < 0.05)

Our next studies aimed to determine whether Sp1 is a major regulator of FST transcription in cav-1 KO MC. We first assessed if Sp1 overexpression can increase the transcriptional activity of the -123 bp mouse FST promoter. Figure 4a shows that the overexpressed Sp1 is functionally active, effectively increasing Sp1 transcriptional activity. In cav-1 WT MC, Sp1 overexpression also significantly increased FST promoter transcriptional activity (Fig. 4b), showing a prominent role for Sp1 in FST promoter regulation. We then downregulated Sp1 using siRNA to assess whether it is essential for the increased FST seen in cav-1 KO MC. Figure 4c confirms the successful knockdown of Sp1. As we hypothesized, Sp1 knockdown reduced FST promoter transcriptional activity in KO MC to levels seen in WT cells (Fig. 4d). FST mRNA and protein expression were similarly reduced (Fig. 4e, f). A smaller decrease in protein levels was also observed in cav-1 WT cells, demonstrating the importance of Sp1 to basal FST regulation.

We next wished to confirm that Sp1 binds to the FST − 123 promoter region. Using ChIP coupled with qRT-PCR, we quantified the amount of Sp1 binding to the putative Sp1 binding sites localized within the -123 bp promoter region of FST. As seen in Fig. 4g, Sp1 binding in this region was significantly more abundant in cav-1 KO compared to WT MC. To confirm that this is required for transcriptional regulation of FST, we deleted the two identified Sp1 binding sites within the -123 bp region of the FST promoter (Fig. 4h). Deletion of both of these resulted in a significant decrease in FST promoter activity in both cav-1 WT and KO MC. Importantly, the elevated FST promoter activity observed in cav-1 KO compared with WT MC was abolished, highlighting the central role for Sp1 in FST transcriptional regulation by cav-1 (Fig. 4i). The decrease seen in WT MC additionally illustrates the importance of Sp1 to basal FST regulation. Taken together, these data show an important regulatory role for cav-1 in Sp1 expression and transcriptional activity, which leads to a significant upregulation of FST in cav-1 KO cells.

### Cav-1 regulates Sp1 activity through PI3K and PKCζ

**Fig. 5:**
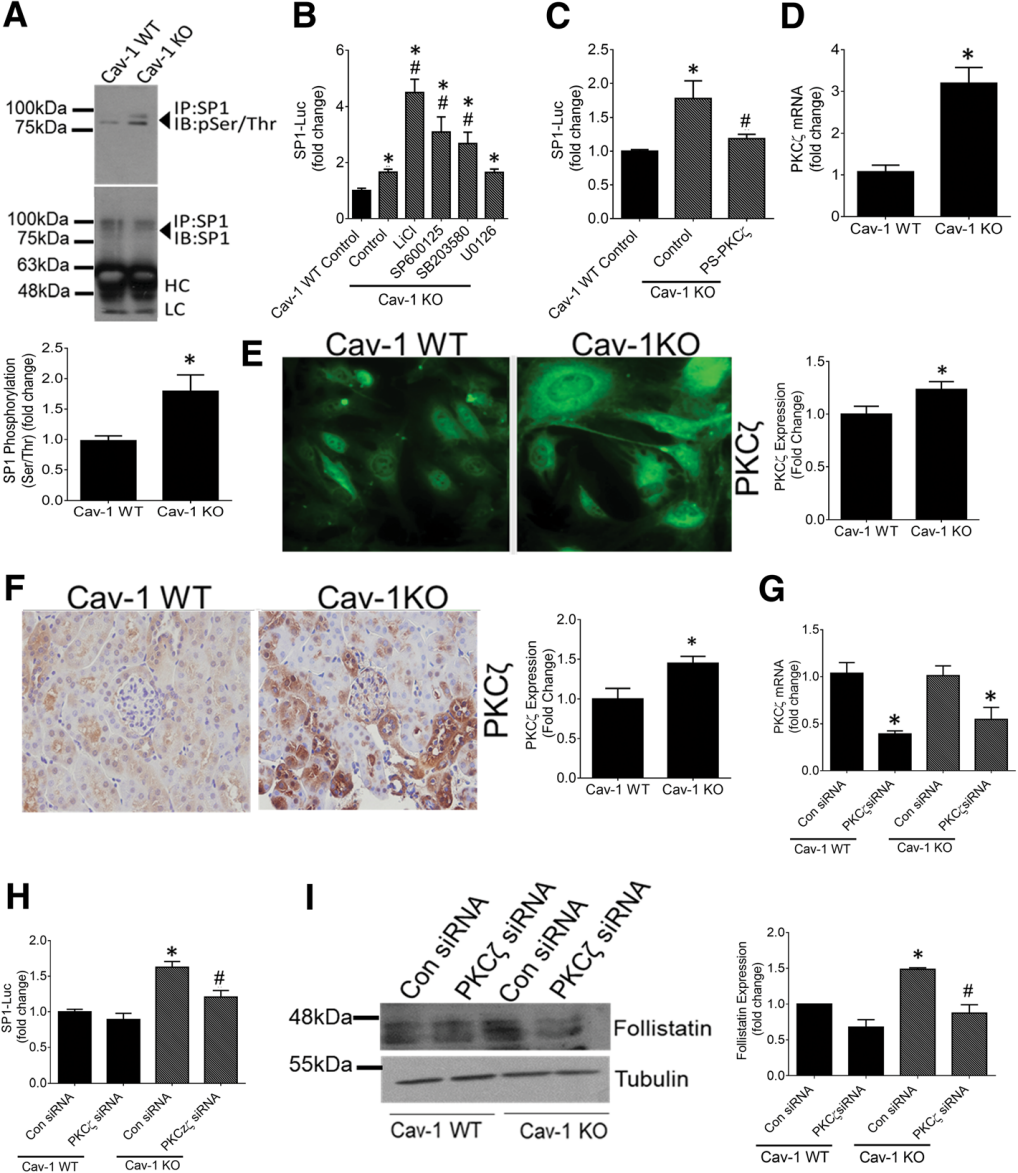
Increased PKCζ induces Sp1 activity to upregulate FST in cav-1 KO MC. **a** Sp1 was immunoprecipitated from cav-1 WT and KO MC and immunoblotted for serine/threonine phosphorylation. Elevated phosphorylation was seen in KO cells (*n* = 3, representative blots shown). **b** None of the following kinase inhibitors reduced Sp1 activity, assessed using the Sp1 reporter construct, to that seen in WT cells: GSK3β inhibitor LiCl (10 mM), JNK inhibitor SP600125 (20 μM), p38 inhibitor SB203580 (5 μM) or MEK/Erk inhibitor U0126 (10 μM) for 24 h. (*n* = 3–6, *vs WT, #vs KO control, *p* < 0.05). **c** PKCζ inhibition with a pseudo-substrate inhibitor peptide (PS-PKCζ) (10 μM, 24 h) abolished the increased Sp1 activity in cav-1 KO MC (*n* = 9, *vs WT, # vs KO control, *p* < 0.05). **d** PKCζ mRNA (*n* = 3, *p < 0.05) and (**e**) protein expression (*n* = 2, *p < 0.05, representative micrographs shown) was significantly elevated in cav-1 KO MC. **f** PKCζ expression was elevated in the kidneys of cav-1 KO mice in both tubules and glomeruli (*n* = 3 mice, *p < 0.05, representative micrographs shown). **g** Effective siRNA-mediated PKCζ knockdown was confirmed by qRT-PCR in cav-1 WT and KO MC (*n* = 3,*p < 0.05 vs con siRNA for both WT and KO MC normalized to their own controls). **h**, **i** PKCζ knockdown reduced both the increased Sp1 activity (**h**) and FST protein expression (**i**) in KO MC to levels seen in WT cells (G: *n* = 9, *vs WT con siRNA, # vs KO con siRNA, *p* < 0.05; H: *n* = 3, *vs WT con siRNA, #vs KO con siRNA, *p* < 0.05)

The regulation of Sp1 by cav-1 has not previously been described. We thus wanted to determine the mechanism underlying this observation. It is well known that Sp1 activity is under tight regulation via phosphorylation, which can either positively or negatively influence the activity and binding of Sp1 to its downstream targets [29]. Since serine/threonine (ser/thr) phosphorylation of Sp1 by various kinases was shown to be an important stimulator of Sp1activity, we compared baseline Sp1 ser/thr phosphorylation between cav-1 KO and WT cells. After Sp1 was immunoprecipitated from total cell lysate, immunoblotting for phosphorylated ser/thr sites showed a greater degree of Sp1 ser/thr phosphorylation in cav-1 KO MC (Fig. 5a).

Next, we sought to identify the kinase responsible for increased Sp1 phosphorylation in cav-1 KO MC. Using various inhibitors, we screened kinases known to phosphorylate Sp1 at ser/thr residues and thereby enhance Sp1 activity. Figure 5b shows that in cav-1 KO MC, glycogen synthase kinase 3 beta (GSKβ) (using LiCl), c-Jun N-terminal kinase (JNK) (using SP600125) and p38 mitogen-activated protein kinase (MAPK) (using SB203580) inhibition significantly upregulated Sp1 activity, while mitogen-activated protein kinase kinase (MEK) inhibition (using U0126) did not affect Sp1 activity. None of these kinases are thus responsible for the upregulation of Sp1 activity in cav-1 KO MC.

We next assessed the role of protein kinase C zeta (PKCζ), which has been shown in numerous cell types to phosphorylate and positively regulate Sp1 activity [30]. We found that PKCζ inhibition using a PKCζ pseudosubstrate reduced the increased activity of Sp1 in cav-1 KO MC to WT levels (Fig. 5c). We then questioned whether the expression of PKCζ is altered in cav-1 deficient MC. Interestingly, both PKCζ mRNA (Fig. 5d) and protein (Fig. 5e) were significantly increased in cav-1 KO MC. Upregulation of PKCζ expression was also observed in the glomeruli and tubules of cav-1 KO mice (Fig. 5f). To further confirm the role of PKCζ as an upstream regulator of the increased Sp1 activity seen in cav-1 KO cells, we downregulated PKCζ using siRNA. Figure 5g shows effective PKCζ knockdown in cav-1 WT and KO MC. Similar to pharmacologic inhibition, this reduced Sp1 activity in cav-1 KO MC to levels seen in WT cells (Fig. 5h). Accompanying decreases in FST protein expression were also seen (Fig. 5i).

**Fig. 6:**
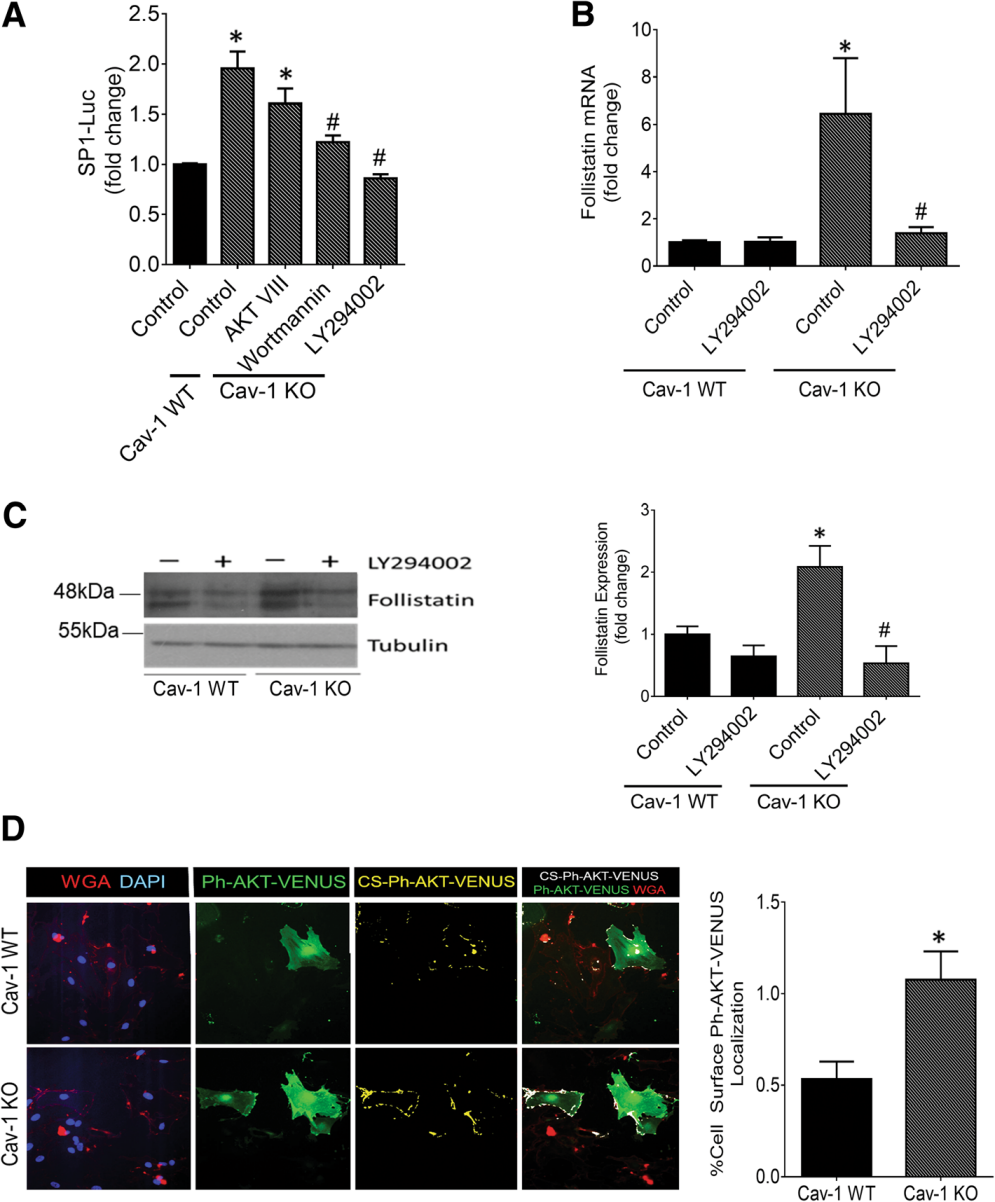
PI3K is a caveolin-1-regulated mediator of PKCζ required for FST upregulation in cav-1 KO MC. **a** The PI3K inhibitors wortmannin (500 nM) or LY294002 (20 μM), but not the Akt inhibitor Akt VIII (20μM) for 24 h abolished the increased Sp1 activity observed in cav-1 KO vs WT MC (*n* = 6, *vs WT, # vs KO control, *p* < 0.05). **b** PI3K inhibition prevented the increase in FST mRNA expression observed in cav-1 KO MC (*n* = 5, *vs WT, #vs KO control, *p* < 0.05). **c** PI3K inhibition also abolished the increased FST protein expression observed in cav-1 KO vs WT MC (*n* = 4, *vs WT, #vs KO control, *p* < 0.05). **d** Cav-1 WT and KO MC were transfected with the fluorescent PIP3 biosensor PH AKT-Venus (green). Elevated basal PI3K activity, as observed by increased PIP3 production at the plasma membrane, delineated by WGA (red), was seen in cav-1 KO MC. This is highlighted by the white co-localization mask (*n* = 3, 21 micrographs quantified, with representative micrographs shown)

PI3K was shown to be an important promoter of PKCζ activity in numerous cell types [30]. To assess the importance of PI3K-mediated activation of PKCζ in Sp1 activation and thereby FST regulation in cav-1 KO MC, we examined the effects of two distinct PI3K inhibitors. Both of these (wortmannin and LY294002) significantly reduced Sp1 activity in cav-1 KO MC to that seen in WT cells (Fig. 6a), although LY249002 was more efficacious than wortmannin in this regard. This may be due to the greater stability of LY294002 in solution [31]*.* Akt is a ser/thr kinase well-known to be activated by PI3K [32]. However, the Akt inhibitor VIII did not reduce Sp1 activity in KO cells. This is in keeping with a known role for the enzyme phosphoinositide-dependent protein kinase-1 (PDK-1) and not Akt as the kinase downstream of PI3K which phosphorylates and activates PKCζ [33].

**Fig. 7:**
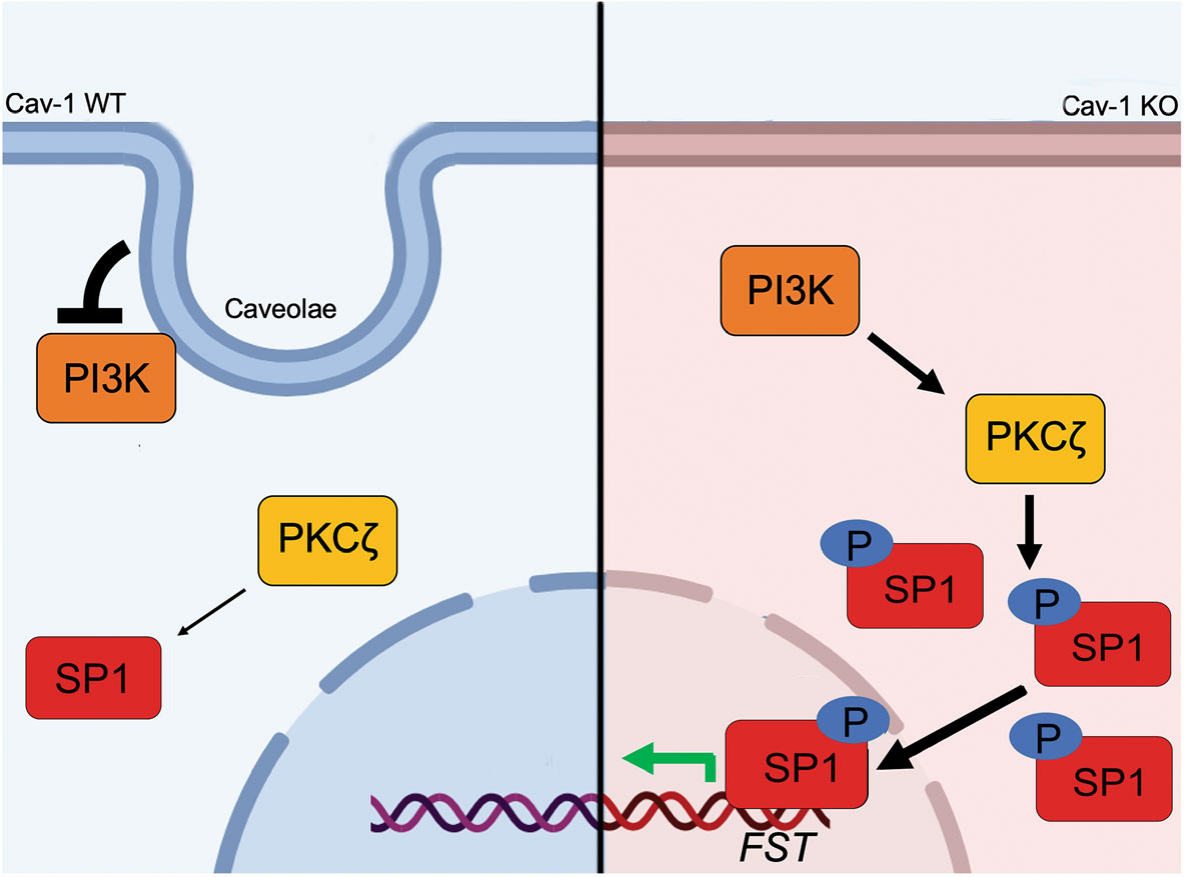
Proposed molecular mechanism for the regulation of FST by cav-1. In the absence of cav-1/caveolae, enhanced PI3K activity activates PKCζ which augments Sp1 phosphorylation, nuclear accumulation and activity. This increases Sp1 binding to the -123 bp region of the FST promoter, resulting in elevated FST transcriptional activation and protein expression

We next tested the effects of PI3K inhibition on FST mRNA and protein expression. PI3K inhibition significantly reduced FST mRNA expression in cav-1 KO MC (Fig. 6b). Figure 6c similarly shows that, in cav-1 KO MC, PI3K inhibition reduced FST protein expression to levels seen in WT cells. Last, we assessed whether cav-1 KO MC have increased PI3K activity. Here, we transfected cav-1 WT and KO MC with a fluorescent biosensor (Ph-Akt-Venus) for phosphatidylinositol 3,4,5-trisphosphate (PIP3) [21]. Class I PI3Ks are responsible for the production of PIP3 at the plasma membrane. Thus, the translocation of the pleckstrin homology (Ph) domain of Akt to the plasma membrane is indicative of PIP3 generation and can serve as a readout of enzymatic PI3K activity. A fluorophore-labeled wheat germ agglutinin (WGA) was used to label the plasma membrane to confirm localization. Figure 6d shows a pronounced increase in PI3K activity in cav-1 KO MC, identified by colocalization of Ph-Akt-VENUS and WGA and highlighted using a colocalization mask (seen in white). Quantification of the colocalization mask is shown in the accompanying graph. Collectively, as summarized in Fig. 7, our results show that cav-1 deficient MC exhibit increased activity of PI3K, an upstream regulator of PKCζ activity. Increased PKCζ activity results in elevated Sp1 activation which augments FST transcription.

## Discussion

Our previous studies have identified a critical role for cav-1 in the ability of MC to produce extracellular matrix proteins and profibrotic cytokines, both basally and in response to profibrotic stimuli [11–13]. Increased cav-1 expression has been observed in several fibrotic kidney diseases in both animal models and humans [14, 34–36]. Furthermore, studies using cav-1 knockout mice and cav-1 deficient cells have shown that the elimination of cav-1 can protect against fibrosis both in vivo and in vitro, while having no adverse effects on blood pressure and renal function [10, 11, 13, 37]. As of now, targeting cav-1 via therapeutic approaches has not been feasible. In our efforts to better understand how cav-1 deficient MC are protected against the profibrotic effects of several stimuli relevant to chronic kidney disease such as high glucose, TGFβ1 and mechanical stress [11–13], we identified significant upregulation of the antifibrotic protein FST in mouse MC lacking cav-1. Functionally, we show that inhibiting the expression of FST in cav-1 KO MC restored matrix production both basally and in response to profibrotic stimuli and that supplementing exogenous FST in cav-1 WT MC protected against matrix protien production. We also identified the mechanism by which cav-1 deficiency led to FST upregulation. Our data now identify a novel role for cav-1 in controlling activity of the transcription factor Sp1, a critical regulator of FST transcription in MC. We further provide mechanistic insight into Sp1 regulation by cav-1, showing that this occurs at the post-translational level through control of PI3K-PKCζ signaling. Figure 7 highlights our proposed molecular mechanism through which cav-1 regulates expression of the antifibrotic protein FST in glomerular mesangial cells. These findings carry important implications for the potential use of therapies targeting this pathway in the treatment of chronic kidney disease, as discussed below.

Interestingly, a related follistatin-domain containing protein, follistatin-like 3 (FSTL-3), has been shown to protect against matrix production in MC exposed to high glucose [38]. FSTL-3 functions similarly to FST, binding and neutralizing similar TGFβ superfamily family members. However, it is distinct from FST due to the absence of a heparin binding motif, preventing its binding to cell surface heparan-sulfate proteoglycans as occurs with FST [39, 40]. Thus, differences in in vivo biologic activities between FSTL-3 and FST likely exist. We did not find any differences in FSTL-3 transcript expression in cav-1 WT and KO MC (Additional file 3: Figure S2), thus excluding a major contribution of FSTL-3 to the antifibrotic phenotype we observed in cav-1 KO MC.

Several transcription factors have thus far been found to regulate FST expression in different cell types, including CREB, Smad3 and β-catenin [28, 41–43]. Sp1, a ubiquitously expressed transcription factor, was also noted to activate the FST promoter in intestinal epithelial cells [28]. Our findings in MC support an important role for Sp1 in regulation of the FST promoter, and further identify elevated Sp1 activity as the mechanism by which cav-1 deletion leads to FST upregulation. Interestingly, we found that only a very short segment (123 bp) of the proximal promoter, containing two Sp1 binding sites, regulates FST promoter activity both basally and in response to cav-1 deletion. Concurrent with our findings, a region 262 bp upstream of the translation start site was previously shown to be critical for the regulation of FST expression in a manner reflecting endogenous mRNA expression [26].

How Sp1 activity is regulated by cav-1 is not as yet understood. It is well known, however, that it is under tight regulation via numerous post-translational modifications such as phosphorylation, acetylation, sumoylation, ubiquitylation, and glycosylation [29]. These can positively or negatively influence Sp1 DNA binding and activity [29]. Since cav-1 is a well-known regulator of a wide variety of intracellular signaling cascades, we initially assessed whether cav-1 deficiency altered Sp1 phosphorylation, the most well described Sp1 post-translational modification. Our results now reveal novel regulation of Sp1 ser/thr phosphorylation, and hence activation, by cav-1. In seeking to identify the mechanism behind this increased phosphorylation, we further identified augmented activity of PI3K-PKCζ signaling in cav-1 deficient MC as a mediator of this increased Sp1 phosphorylation.

PI3K is a lipid kinase that catalyzes the formation of a family of phosphoinositides, including PIP3, with an important role in cell growth and transformation [44, 45]. Our studies illustrate that cav-1 deficient MC exhibit basally elevated PI3K activity and signaling compared to cav-1 WT cells. Although a few studies have assessed the regulation of PI3K by cav-1, these show discordant effects, likely dependent on cell type. Thus, in cancer cells (HeLa), cav-1 overexpression increased PI3K activity [46], while in fibroblasts the opposite effects were observed [47]. In some studies, while PI3K activity was not assessed directly, downstream signaling was found to be regulated by cav-1. For example, in hepatoma cells, increased cav-1 expression induced by plasmalogens was associated with decreased Akt activity, suggesting inhibitory effects of cav-1 on PI3K [48]. The inhibitory association of cav-1 with p85, the regulatory subunit of PI3K, was suggested to mediate this effect [47]. However, inhibition of PI3K signaling by cav-1 may also occur through an indirect mechanism by augmenting activity of PTEN (phosphatase and tensin homolog). This lipid phosphatase acts as the primary suppressor of PI3K signaling by dephosphorylating PIP3 [49]. Xia et al. demonstrated that cav-1 deficient fibroblasts have lower PTEN activity than their WT counterparts. Cav-1 reconstitution increased PTEN membrane localization and activity, and this was associated with a reduction in signaling downstream of PI3K (Akt activity) [50]. Like PI3K, PTEN was also found to physically interact with cav-1 [50]. These data thus suggest both direct and indirect regulation of PI3K by cav-1, and support our findings that cav-1 represses PI3K activity.

The most well studied mediator of PI3K signaling is the ser/thr kinase Akt. Our data, however, excluded a role for Akt in FST regulation by cav-1. Instead, we identified PKCζ, also known to function downstream of PI3K, as the effector for Sp1 activation and FST upregulation in cav-1 deficient MC. PKCζ is a ser/thr kinase which activates Sp1 through phosphorylation of residues in its zinc region [29, 51, 52]. Unlike most other PKC isoforms, PKCζ is activated without the need for calcium and/or diacylglycerol [53]. Secondary messenger lipids including PIP3 recruit PKCζ from cytosol to the membrane through binding to its regulatory domain. They can also induce a conformational change that removes auto-inhibition of PKCζ catalytic activity [53, 54]. At the membrane, the PI3K-dependent kinase PDK-1 can activate PKCζ through phosphorylation on its activation loop [33]. Similar to several other, but not all, PKC isoforms, PKCζ was shown to interact with the cav-1 scaffolding domain, a region of cav-1 that mediates its interaction with numerous other proteins [55, 56]. This interaction was also found to inhibit PKCζ autophosphorylation and kinase activity [55]. Furthermore, PDK1 interaction with cav-1, which reduced its kinase activity, was also found [57]. Cav-1/caveolae thus function at several levels to inhibit PKCζ activation. In disagreement with this, however, PKCζ localization to caveolae was associated with increased activity in response to the lipid metabolite ceramide, highlighting stimulus specificity for the role of cav-1/caveolae in PKCζ activation [58]. Finally, it should be noted that we also observed elevated PKCζ transcript levels in cav-1 deficient MC. The mechanism underlying this will be defined in future studies.

Interestingly, while pharmacologic PI3K and PKCζ inhibition blunted Sp1 activity, GSK, JNK and p38 inhibition in our studies was found to promote Sp1 activity. In agreement with these findings, these kinases have been individually shown to negatively regulate components of the PI3K/PKCζ pathways, which converge to modulate Sp1 activity. For example, in murine microglia cells GSK3 inhibition increased nuclear Sp1 expression and activity along with increased IL-10 production through elevated PI3K activity [59]. In chondrocytes, p38 was shown to bind the regulatory domain of PKCζ, preventing its autophosphorylation and thereby inhibiting its activity [60]. Finally, in human lung cancer cells, JNK inhibited Sp1 and thereby its downstream target genes that regulate cell growth [61]. However, in some settings, positive regulation of PI3K/PKCζ/Sp1 by JNK has been found. For example, in lung epithelial cells JNK increased Sp1 phosphorylation and activity in repose to oxidative stress [62]. In another study, JNK was found to positively regulate PKCζ through affecting its localization to podosomes [63]. These differences could be attributed to differences in the cell type and stimulus being investigated. Nonetheless, it is likely that these kinases function through modulation of PI3K/PKCζ to affect Sp1 transcriptional activity.

Collectively, our data have thus identified novel regulation of FST transcription by cav-1/caveolae through suppression of PI3K/PKCζ/Sp1 signaling. It is noteworthy that in several fibrotic kidney diseases in both rodent models and humans, renal cav-1 expression is elevated [14, 34–36]. This would be expected to attenuate FST expression, thereby inhibiting its protective antifibrotic effect. Therapies to increase activity of Sp1 or its upstream mediators would thus seem to be of potential therapeutic interest. However, activation of PI3K, PKCζ and Sp1 have all also been associated with renal profibrotic effects [64–66]. Indeed, Sp1 was shown to activate the transcription of several profibrotic and matrix protein genes including PAI-1,TGFβ1, fibronectin, PDGF-BB, α-SMA and collagen in various cell types [64, 67–69]. Sp1 was also shown to act synergistically with profibrotic signaling molecules such as Smad3 in response to TGFβ1 to promote matrix production [70]. Furthermore, inhibition of Sp1 activity using ring-type Sp1 decoy oligonucleotides attenuated kidney fibrosis in the unilateral obstruction model [71]. While these data support a profibrotic role for Sp1, it should be noted that Sp1 also regulates a concurrent protective response to limit the extent of fibrosis. Thus, Sp1 mediates induction of the antifibrotic protein Smad7 by TGFβ1 [72], and as our data show, of the antifibrotic protein FST.

Taken together, our data show a novel role for cav-1 in the post-translational regulation of Sp1 through PI3K/PKCζ signaling. Importantly, we established that Sp1, which has thus far been identified as a profibrotic factor in kidney disease, is a critical transcriptional regulator for the antifibrotic protein FST. Thus, therapeutically targeting enhanced activity of PI3K/PKCζ/Sp1 is not a viable option for the treatment of kidney disease due to potential unwanted profibrotic effects. Future studies should further address the therapeutic potential of FST administration in the treatment of fibrotic kidney disease.

## Conclusion

Our results identified Sp1 as the critical transcription factor regulating activation of the FST promoter in MC lacking cav-1 through binding to a region within 123 bp of the transcription start site. Absence of cav-1 increases Sp1 transcriptional activity through augmented activation of phosphoinositide 3-kinase (PI3K) and its downstream mediator protein kinase C (PKC) zeta. In turn, PKC zeta phosphorylates and activates Sp1. These findings describe a novel transcriptional mechanism regulated by cav-1 which functions to repress the expression of FST, a major antifibrotic protein. These findings provide important knowledge that will inform the development of antifibrotic treatment strategies for chronic kidney disease.

## Additional files

Additional file 1: **Table S1.** Drugs. **Table S2.** Plasmids and siRNA. **Table S3.** Antibodies. **Table S4.** qPCR primers. Table S5. Cloning Sequences. (DOCX 23 kb)

Additional file 2: **Figure S1.** (A) Exogenous recombinant FST (1 μg/ml) protects against TGFβ1 (0.5 ng, 24 h)-induced extracellular matrix (ECM) production in cav-1 WT MC (*n* = 2). (B) siRNA (50 nM)-mediated FST downregulation augments TGFβ1 (0.5 ng, 24 h)-induced ECM production in cav-1 KO MC (*n* = 2). (TIF 29033 kb)

Additional file 3: **Figure S2.** The mRNA expression of FSTL-3 is not significantly different between cav-1 WT and KO MC (*n* = 6). (TIF 13489 kb)

## Abbreviations

Cav-1: Caveolin-1
FST: Follistatin
KO: Knockout
MC: Mesangial cells
PDK-1: Phosphoinositide-dependent protein kinase-1
PH: Pleckstrin homology
PI3K: Phosphoinositide 3-kinase
PIP3: Phosphatidylinositol 3,4,5-trisphosphate
PKCζ: Protein kinase C zeta
PTEN: Phosphatase and tensin homolog
ser/thr: Serine/threonine
TGFβ: Transforming growth factor beta
TSS: Transcription start site
WGA: Wheat germ agglutinin
WT: Wild-type
