# Supplementary material for: Caveolin-1 regulation of Sp1 controls production of the antifibrotic protein follistatin in kidney mesangial cells
Source: Cell Commun Signal. 2019 Apr 17;17:37. doi: 10.1186/s12964-019-0351-5 (PMC6472091; doi:10.1186/s12964-019-0351-5)
Supplement: Supplementary file 1 — Table S1. Drugs. Table S2. Plasmids and siRNA. Table S3. Antibodies. Table S4. qPCR primers. Table S5. Cloning Sequences. (DOCX 23 kb) [file 12964_2019_351_MOESM1_ESM.docx]

**Table S1.** Drugs

| ***Drug*** | ***Dose*** | ***Source*** |
| --- | --- | --- |
| **Cycloheximide** | 10µg/ml | Sigma |
| **Actinomycin D** | 1µg/ml | Sigma |
| **PS-PKC ζ (pseudosubstrate)** | 10µM | Tocris |
| **Wortmannin** | 500nM | Sigma |
| **LY-294002** | 20µM | Sigma |
| **LiCl** | 10mM | Sigma |
| **AKT VIII** | 20µM | EMD |
| **U0126** | 10µM | Promega |
| **SB203580** | 5µM | Sigma |
| **SP600125** | 20µM | EMD |
| **Follistatin** | 1µg/ml | R&D Systems |

**Table S2.** Plasmids and siRNA

| ***siRNA or Plasmid*** | ***Amount*** | ***Source*** |
| --- | --- | --- |
| **Mouse SP1 ON Target Smart Pool siRNA** | 50nM | Dharmacon |
| **Mouse PKCζ ON Target Smart Pool siRNA** | 100nM | Dharmacon |
| **Mouse Cav-1 Silencer Select SiRNA** | 150nM | Life Tech |
| **Mouse Follistatin Silencer Select SiRNA** | 50nM | Life Tech |
| **On-target plus siCONTROL non-targeting siRNA** | 50nM | Dharmacon |
| **Control Silencer Select SiRNA** | 50nM | Life Tech |
| **mFST4-FL Luciferase** |  | Dr. Jeong Yoon |
| **mFST4Δintron1-1840 (mFST-4 Luciferase)** |  | Generated in lab |
| **mFST4Δintron1-1380** |  | Generated in lab |
| **mFST4Δintron1-915** |  | Generated in lab |
| **mFST4Δintron1-520** |  | Generated in lab |
| **mFST4Δintron1-244** |  | Generated in lab |
| **mFST4Δintron1-123** |  | Generated in lab |
| **mFST4Δintron1-123 ΔSP1** |  | Generated in lab |
| **3xmt SP1 Luciferase** |  | Dr. Peter Di |
| **pCMV β-galactosidase** |  | Clonetech |
| **CMV-GST-SP1-HA** |  | Dr. Jane Clifford |
| **PH Akt-Venus** |  | Dr. Narasimhan Gautam (Addgene plasmid # 85223) |
| **pcDNA3.1(+) (plasmid)** |  | Thermo Scientific |

**Table S3.** Antibodies

| *Antibody* | *Application* | *Dilution/Amount* | *Source* |
| --- | --- | --- | --- |
| Caveolin-1 | WB | 1:1,000 | BD Biosciences; 610059 |
| Follistatin | WB | 1:1,000 | Santa Cruz; sc-30194 |
| Follistatin | IHC | 1:100 | Proteintech ; 60060-1-Ig |
| SP1 | WB | 1:1,000 | Pierce; 82406 |
| SP1 | CHIP/IP and IHC/IF | 1µg and 1:500 | Abcam; ab13370 |
| Mouse IgG | IP | 1µg | Millipore; 12371 |
| pSerine/threonine | WB | 1:1000 | BD Transduction; 612548 |
| PKCζ | IHC and IF | 1:100 and 1:50 | Santa Cruz; sc-393218 |
| α-Tubulin | WB | 1:10,000 | Sigma; T6074 |
|  |  |  |  |

**Table S4.** qPCR primers

| ***Gene*** | ***Forward*** | ***Reverse*** |
| --- | --- | --- |
| **FST** | AAAACCTACCGCAACGAATG | GGTCTGATCCACCACACAAG |
| **SP1–FST-123bp (CHIP)** | TCACCTGATTCACACTGAAC | TTCAATGGACGTCAGAAGCC |
| **PKCζ** | GCCTCCCTTCCAGCCCCAGA | CACGGACTCCTCAGCAGACAGCA |
| **FSTL3** | ACTCTGTGGCAACAACAACG | TTCTCTTCCTCCTCTGCTGG |
| **18s** | GCCGCTAGAGGTGAAATTCTTG | CATTCTTGGCAAATGCTTTCG |

**Table S5.** Cloning Sequences

| ***Construct*** | ***Forward*** | | ***Reverse*** |
| --- | --- | --- | --- |
| **mFST4 Δintron1 -1840** | CATGGTACCAGATTAAGAAGGATGTGAAG | | CATGCTAGC CGCGCGATTCAATGGACGTC |
| **mFST4 Δintron1 -1380** | CATGGTACCGGGCTAGAGAAGAAGGGCGA | | CATGCTAGC CGCGCGATTCAATGGACGTC |
| **mFST4 Δintron1 -915** | CATGGTACCGCGACGAAGTGAAAGGGGAG | | CATGCTAGC CGCGCGATTCAATGGACGTC |
| **mFST4 Δintron1-520** | CATGGTACCAGAGGTGCTGGGGACCCAT | | CATGCTAGC CGCGCGATTCAATGGACGTC |
| **mFST4 Δintron1-244** | CATGGTACCGCCGCTTTGATTTCGGGCAC | | CATGCTAGC CGCGCGATTCAATGGACGTC |
| **mFST4 Δintron1-123** | CATGGTACCTCGGTCGCGGCCGCCCT | | CATGCTAGC CGCGCGATTCAATGGACGTC |
| ***mFST4Δintron1-123ΔSP1*** *(deleted SP1 binding sites are underlined)* | | | |
| ***Forward Strand/Sense*** | | ***Reverse Strand/Anti-Sense*** | |
| CTCGGTCGCGGCCGCCCTCCCACAGCCCCACACACTGGGAGACCGCCCACCGCAAACCTCGGAGACCCCCGTCTAGATTTAAAGCGCGGCTGCGCCCGGCTTCTGACGTCCATTGAATCGCGCG | | CTAGCGCGCGATTCAATGGACGTCAGAAGCCGGGCGCAGCCGCGCTTTAAATCTAGACGGGGGTCTCCGAGGTTTGCGGTGGGCGGTCTCCCAGTGTGTGGGGCTGTGGGAGGGCGGCCGCGACCGAGGTAC | |
