# Supplementary figures and images for: Caveolin-1 regulation of Sp1 controls production of the antifibrotic protein follistatin in kidney mesangial cells
Source: Cell Commun Signal. 2019 Apr 17;17:37. doi: 10.1186/s12964-019-0351-5 (PMC6472091; doi:10.1186/s12964-019-0351-5)

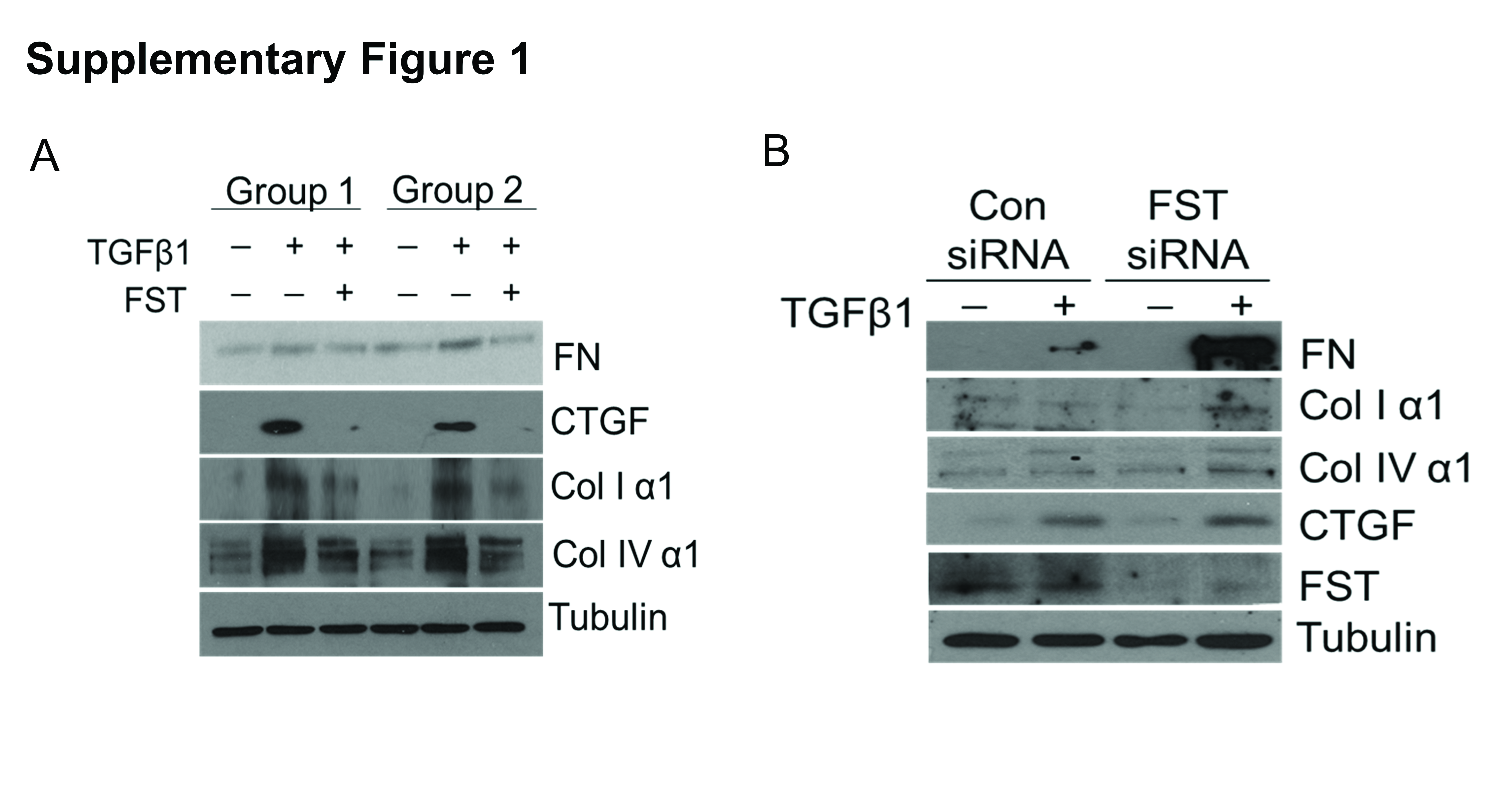

Supplement: Supplementary file 2 — Figure S1. (A) Exogenous recombinant FST (1 μg/ml) protects against TGFβ1 (0.5 ng, 24 h)-induced extracellular matrix (ECM) production in cav-1 WT MC (n = 2). (B) siRNA (50 nM)-mediated FST downregulation augments TGFβ1 (0.5 ng, 24 h)-induced ECM production in cav-1 KO MC (n = 2). (TIF 29033 kb) [file 12964_2019_351_MOESM2_ESM.tif]

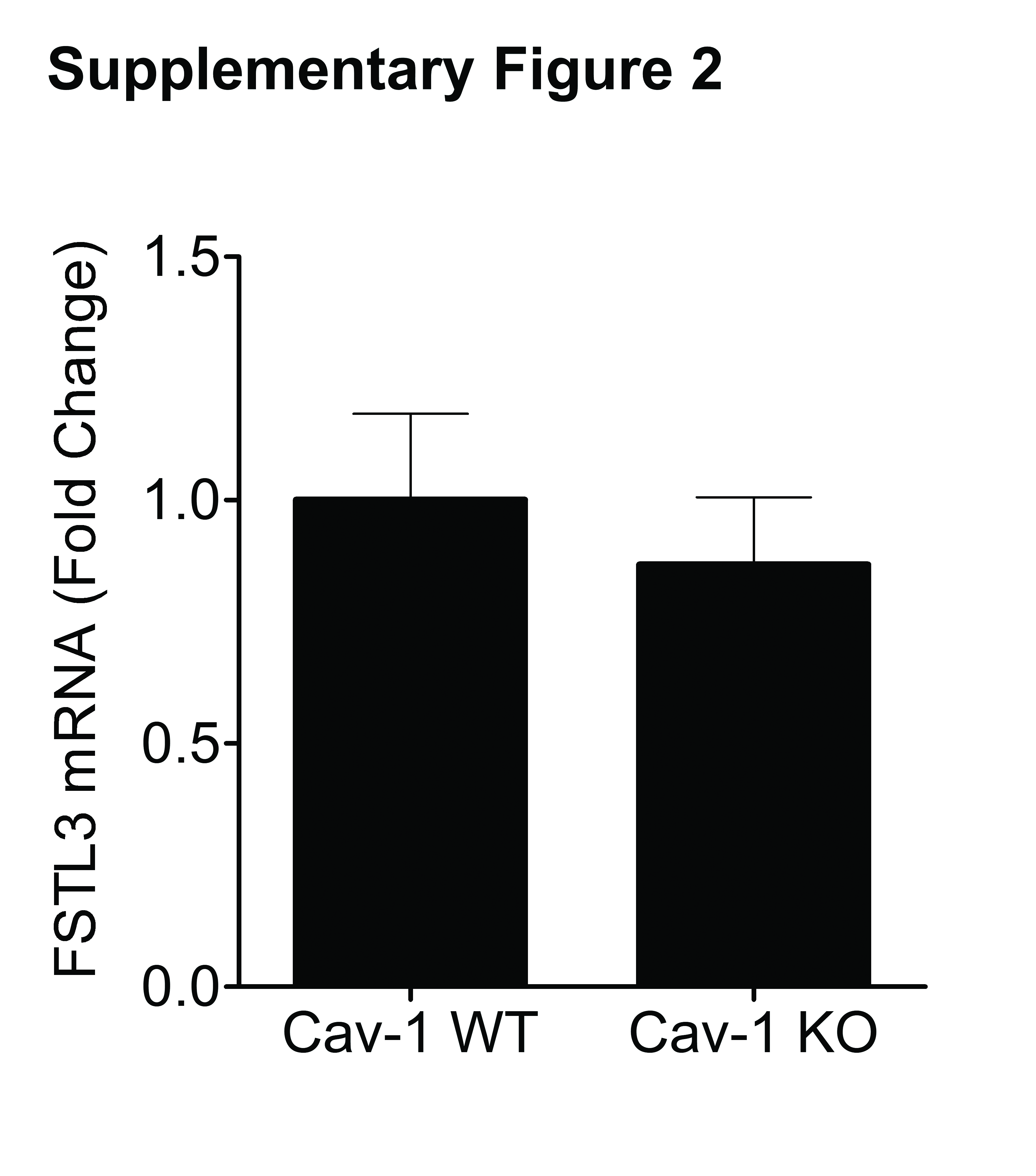

Supplement: Supplementary file 3 — Figure S2. The mRNA expression of FSTL-3 is not significantly different between cav-1 WT and KO MC (n = 6). (TIF 13489 kb) [file 12964_2019_351_MOESM3_ESM.tif]
